# Flow Mediated Dilatation Is Reduced with the Progressive Stages of Glomerular Filtration Rate and Albuminuria in Type 2 Diabetic Patients without Coronary Heart Disease

**DOI:** 10.1155/2015/728127

**Published:** 2015-04-29

**Authors:** Hiroyuki Ito, Mina Nakashima, Kentaro Meguro, Haruki Furukawa, Hitomi Yamashita, Akifusa Takaki, Chizuko Yukawa, Takashi Omoto, Masahiro Shinozaki, Shinya Nishio, Mariko Abe, Shinichi Antoku, Mizuo Mifune, Michiko Togane

**Affiliations:** ^1^Department of Diabetes, Metabolism and Kidney Disease, Edogawa Hospital, 2-24-18 Higashikoiwa, Edogawa, Tokyo 133-0052, Japan; ^2^Laboratory Department, Edogawa Hospital, 2-24-18 Higashikoiwa, Edogawa, Tokyo 133-0052, Japan; ^3^Department of Cardiology, Edogawa Hospital, 2-24-18 Higashikoiwa, Edogawa, Tokyo 133-0052, Japan

## Abstract

We aimed to clarify the usefulness of measuring the flow mediated dilatation (FMD) in patients with type 2 diabetes mellitus without and with coronary heart disease (CHD). The FMD was measured in 480 patients with type 2 diabetes and in 240 nondiabetic subjects. The FMD was significantly lower in the subjects with CHD (*n* = 145, 5.4 ± 3.2%) than in those without CHD (*n* = 95, 6.9 ± 3.5%) among the nondiabetic subjects. The FMD was also lower in the subjects both with CHD (*n* = 161, 5.6 ± 2.8%) and without CHD (*n* = 319, 6.1 ± 3.3%) among the patients with diabetes compared to those without both diabetes and CHD. The FMD showed a significant positive correlation with the estimated glomerular filtration rate (eGFR) in the diabetic patients without CHD, while there was no significant association in those with CHD. The FMD was significantly lower with the progressive stages of the GFR or albuminuria in the patients without CHD among those with diabetes, although the FMD was not different in those with CHD. In conclusion, the FMD is considered to be useful for the detection of atherosclerosis in patients with type 2 diabetes, even if overt macroangiopathy is not diagnosed.

## 1. Introduction

Diabetic macroangiopathies affect the prognosis and quality of life of the patients with type 2 diabetes mellitus. Several surrogate markers for atherosclerosis, such as the ankle-brachial pressure index (ABI), brachial-ankle pulse wave velocity (baPWV), and carotid intima-media thickness (cIMT), are widely used in the clinical setting and are recognized to be useful for the diagnosis of diabetic macroangiopathies [1–3]. Because these physiological examinations quantify the atherosclerosis that is currently present in the vessel walls, there might be an underdiagnosis in subjects who do not yet have narrowing of the vessel lumen or decreased elasticity of the vessel wall.

The injury of the endothelial cells of the arteries has been reported to be found in the earliest stage of atherosclerosis in individuals with hypertension, dyslipidemia, and/or diabetes mellitus [4, 5]. It is considered that the release of vasodilators, such as nitric oxide (NO), from the arterial endothelium is reduced in conditions associated with atherosclerosis, including patients with diabetic macroangiopathies [6, 7]. Intra-arterial injection of endothelium-derived NO-releasing substances, such as acetylcholine, is one of the methods used to evaluate the endothelial function; however, noninvasive methods have been studied for application in clinical practice [8].

The flow mediated dilatation (FMD) reflects the vasodilatation caused by endothelium-derived NO following reactive hyperemia, which occurs after the recovery from ischemia in the upper limb, and can be used to evaluate the endothelial function by ultrasonographic examination from the body surface. Because the complexity of the procedure has been semiautomated by a computer [9, 10], the FMD has been recognized to be a useful clinical method for detecting the initial development of atherosclerosis [11–13].

It was previously reported that the FMD is lower in the patients with type 2 diabetes mellitus than in normal subjects [14–23]. However, the significance of the decreased value of the FMD has not been established, because the clinical backgrounds of the subjects in the previous investigations varied. The significance of the FMD in the patients with type 2 diabetes mellitus might be different between subjects with diabetic macroangiopathies and those without overt atherosclerosis. In the present study, we aimed to clarify the usefulness of measuring the FMD in the patients with type 2 diabetes mellitus with and without coronary heart disease (CHD), which is one of the most common atherosclerotic complications.

## 2. Subjects and Methods

### 2.1. Subjects

The FMD was measured in 480 Japanese patients with type 2 diabetes mellitus and in 240 age- and sex-matched Japanese subjects without diabetes mellitus (66 ± 12 years old; male subjects, 66%; body mass index (BMI), 23.5 ± 4.0 kg/m^2^; current smoker, 30%; hypertension, 89%; hyper-LDL-cholesterolemia, 50%; hypo-HDL-cholesterolemia, 40%; estimated glomerular filtration rate (eGFR), 68.3 ± 23.7 mL/min/1.73 m^2^) who underwent consecutive treatments in the Department of Diabetes, Metabolism and Kidney Disease and/or the Department of Cardiology of Edogawa Hospital, Tokyo, Japan, between December 2012 and December 2014.

### 2.2. Measurements of Surrogate Markers for Atherosclerosis

The FMD was evaluated using the method described in the previous reports [10, 18, 21, 24, 25]. In brief, the vessel diameter of the brachial artery was measured using the UNEX EF38G (UNEX Corporation, Nagoya, Japan) after the subjects had rested for more than 15 minutes at room temperature (25°C). Subsequently, the cuff was inflated to 50 mmHg above the systolic blood pressure, which was measured in advance, was held for 5 minutes, and then was deflated. The maximum diameter of the blood vessel of the same region obtained 40 to 60 seconds after deflation was recorded. The FMD was calculated as follows: FMD (%) = (maximum diameter − diameter at rest) × 100/diameter at rest. The ABI, baPWV, and cIMT were also measured as described previously [3].

### 2.3. Confounding Factors

The obese individuals were defined as those having a body mass index ≥25.0 kg/m^2^. Hypertension was defined as a systolic blood pressure ≥140 mmHg and/or a diastolic blood pressure ≥90 mmHg. The participants currently using antihypertensive medications were also classified as positive for hypertension. Hyper-LDL-cholesterolemia was defined as either a serum concentration of LDL-cholesterol ≥3.62 mmol/L, or the current use of lipid-lowering agents. Hypo-HDL-cholesterolemia was defined by a serum HDL-cholesterol concentration <1.03 mmol/L. The eGFR was calculated using the formula reported by Matsuo et al. [26], which is recommended by the Japanese Society of Nephrology. The stage of chronic kidney disease (CKD) was diagnosed based on the urinary albumin-to-creatinine ratio (ACR) and eGFR. The stages of albuminuria were graded as A1 (ACR < 30 mg/g/cr), A2 (30 mg/g/cr ≤ ACR < 300 mg/g/cr), or A3 (300 mg/g/cr ≤ ACR) and the GFR stage was graded as G1 (eGFR ≥ 90 mL/min/1.73 m^2^), G2 (90 mL/min/1.73 m^2^ > eGFR ≥ 60 mL/min/1.73 m^2^), G3a (60 mL/min/1.73 m^2^ > eGFR ≥ 45 mL/min/1.73 m^2^), G3b (45 mL/min/1.73 m^2^ > eGFR ≥ 30 mL/min/1.73 m^2^), G4 (30 mL/min/1.73 m^2^ > eGFR ≥ 15 mL/min/1.73 m^2^), or G5 (15 mL/min/1.73 m^2^ > eGFR) according to the classification for chronic kidney disease proposed by the Kidney Disease: Improving Global Outcomes (KDIGO) published in 2011 [27]. Hyperuricemia was defined by serum uric acid levels >416 *μ*mol/L or as patients using allopurinol or febuxostat.

Diabetic retinopathy included simple, preproliferative, and proliferative retinopathy judged according to the results of a funduscopic examination performed by expert ophthalmologists. Diabetic neuropathy was diagnosed by the presence of two or more components among clinical symptoms (bilateral spontaneous pain, hypoesthesia, or paraesthesia of the legs), the absence of ankle tendon reflexes, and decreased vibration sensations using a C128 tuning fork. The diagnosis of CHD was based on a previous history of myocardial infarction, angina pectoris, or interventions after a coronary angiographic examination. The subjects who complained of chest pain alone without undergoing a coronary angiographic examination were not diagnosed with CHD.

## 3. Statistical Analysis

All data are shown as the means ± SD. The Wilcoxon rank sum test, Kruskal-Wallis test, and *χ* ^2^ test were used for among-group comparisons of the continuous and categorical variables, respectively. A least squares method was used to determine the associations of the FMD with the other clinical parameters. Differences with a value of *P* < 0.05 (two-tailed) were considered to be statistically significant. The JMP statistical software package, version 8.0 (SAS Institute, Cary, NC, USA), was used to perform all of the analyses.

## 4. Results

**Figure 1:**
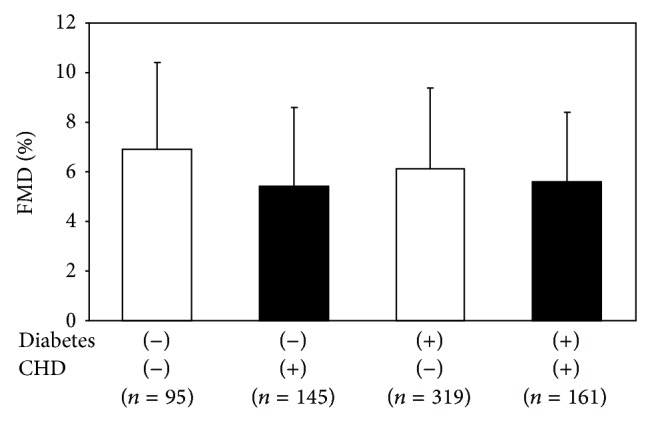
A comparison of the FMD in the groups with and without type 2 diabetes mellitus and CHD.

The FMD in the groups with and without type 2 diabetes mellitus or CHD are shown in Figure 1. The FMD was significantly lower (*P* < 0.01) in the subjects with CHD (*n* = 145, 5.4 ± 3.2%) than in those without CHD (*n* = 95, 6.9 ± 3.5%) among the subjects without diabetes mellitus. The FMD was also lower in the subjects with CHD (*n* = 161, 5.6 ± 2.8%, *P* < 0.01) and those without CHD (*n* = 319, 6.1 ± 3.3%, *P* = 0.07) among the patients with diabetes mellitus than in those without both CHD and diabetes mellitus. The FMD was not significantly different between the subjects with and without CHD among the patients with diabetes mellitus (*P* = 0.17).

Table 1 shows the clinical characteristics of the study subjects with and without type 2 diabetes mellitus. Among the patients with type 2 diabetes, the mean age, duration of diabetes, use of renin-angiotensin system inhibitors or statins, hypertension, hyper-LDL-cholesterolemia, and hypo-HDL-cholesterolemia were significantly higher in the subjects with CHD than in those without CHD. The blood pressure, HbA1c, serum LDL-cholesterol, HDL-cholesterol, eGFR, and ABI were significantly lower in the subjects with CHD than in those without CHD, while the serum uric acid and creatinine concentrations were significantly higher. The cIMT was significantly greater in the subjects with CHD than in those without CHD, while the baPWV did not show any significant difference between the two groups.

**Figure 2:**
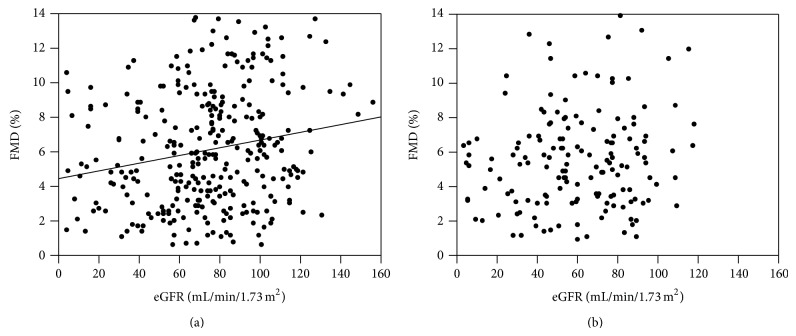
The relationships between the FMD and eGFR in the type 2 diabetic patients (a) without CHD (*n* = 319, *r* = 0.20, *P* < 0.01) and (b) with CHD (*n* = 161, *r* = 0.15, *P* = 0.06).

**Figure 3:**
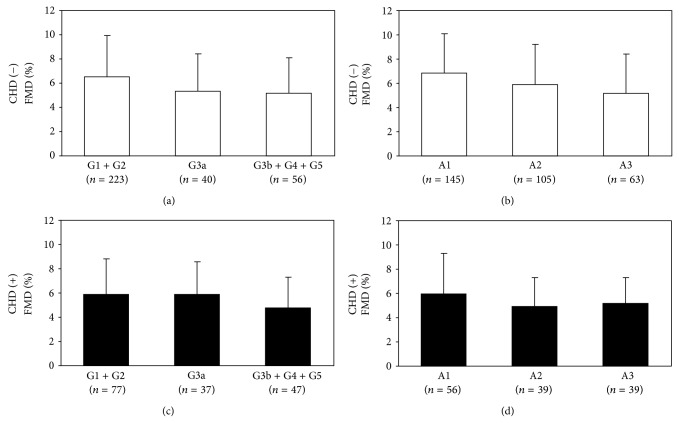
The comparisons of the FMD among (a) GFR stages and (b) albuminuria stages in the patients without CHD and among (c) GFR stages and (d) albuminuria stages in the patients with CHD in the group with type 2 diabetes mellitus.

The FMD showed a significantly positive correlation with the eGFR in the subjects without CHD among the patients with diabetes mellitus (Figure 2(a)), while there was no significant association in those with CHD (Figure 2(b)). The FMD was significantly lower with the progression in the stages of the GFR (*P* < 0.01) or albuminuria (*P* < 0.01) in the patients without CHD (Figures 3(a) and 3(b)), although the FMD was not significantly different based on the GFR (*P* = 0.09) or albuminuria (*P* = 0.42) stages in those with CHD (Figures 3(c) and 3(d)).

Table 2 shows the associations between the FMD and the clinical characteristics of the subjects with and without CHD among the patients with diabetes mellitus. The FMD showed a significantly positive correlation with the eGFR level and a negative correlation with albuminuria in the subjects without CHD, while the FMD was significantly associated with the presence of hypertension and hypo-HDL-cholesterolemia in those with CHD.

## 5. Discussion

It has been described that the FMD is lower in patients with type 2 diabetes mellitus than in nondiabetic subjects [14–23]. In the present study, the FMD was lower in patients with diabetes mellitus, regardless of the presence of CHD, which is one of the diabetic macroangiopathies, compared with subjects without both diabetes mellitus and CHD. Liao et al. also reported that the FMD was lower in the patients with type 2 diabetes mellitus than in healthy controls and that there was no significant difference in the FMD between patients with type 2 diabetes mellitus and nondiabetic patients with stroke or peripheral arterial disease [23]. Although the ABI was significantly lower and the cIMT was significantly higher in the subjects with CHD than in those without CHD among the patients with diabetes mellitus in the present study, the FMD was not significantly different between these two groups. Therefore, it is considered that endothelial dysfunction, which is the earliest stage of atherosclerosis, is potentially present in all patients with type 2 diabetes mellitus, even if there are no obvious macroangiopathies or no abnormalities in the surrogate markers for atherosclerosis, such as the ABI and cIMT. Namely, the patients with type 2 diabetes mellitus might be at a similar risk for atherosclerosis as nondiabetic subjects who have already developed cardiovascular events, regardless of the presence of overt macroangiopathies. The FMD is considered to be especially useful in the diabetic patients who showed no obvious macroangiopathies and no abnormalities in the surrogate markers for atherosclerosis.

In the present study, hypertension and hypo-HDL-cholesterolemia were associated with a low value of the FMD in the patients with type 2 diabetes mellitus and CHD according to a multivariate regression analysis. Because these factors have been recognized as traditional risk factors for atherosclerosis, our results are in agreement with medical common sense. However, it is interesting that only the eGFR and albuminuria were associated with the FMD in the patients without CHD. Recently, it has been clarified that CKD plays an important role in the development of cardiovascular diseases [27, 28]. We have also reported that the presence of CKD in the patients with type 2 diabetes mellitus is associated with an increased number of risk factors for atherosclerosis [29], hypertension resistant to medication [30], surrogate markers for atherosclerosis such as the cIMT [3], lower levels of serum eicosapentaenoic acid/arachidonic acid (which is considered to be a new risk factor for CHD) [31], and high frequencies of diabetic macroangiopathies [32, 33]. The reduction in the FMD with the progressive stages of GFR or albuminuria in the diabetic subjects without CHD may reflect that the presence of CKD is associated with the early stage of atherosclerosis. Therefore, intensive examinations for diabetic macroangiopathies should be considered in the subjects with progressive GFR and albuminuria stages, because it is likely that an endothelial dysfunction is present in these cases, even if overt atherosclerosis is clinically absent.

Many investigators have described the association between the FMD and CKD in patients with type 2 diabetes mellitus. Nair et al. reported that the FMD was lower in 46 patients with type 2 diabetes mellitus than in 20 nondiabetic subjects and that it was lower in the subjects with diabetic nephropathy or retinopathy than in those without [16]. Kawano et al. [24], Yokoyama et al. [34], Makino et al. [35], and Suetsugu et al. [36] showed the associations between the value of the FMD and the subject's age, duration of diabetes mellitus, BMI, blood pressure, insulin resistance, GFR stage, or albuminuria stage in the Japanese subjects with type 2 diabetes mellitus based on investigations without a control group. The FMD was also associated with proteinuria in studies with a small number of subjects performed in China [37] and the USA [38]. However, the frequencies of diabetic macroangiopathies were not described in the studies cited above. Although the Hoorn study performed by Stehouwer et al. reported a reduced FMD in the subjects with macroalbuminuria compared with those with microalbuminuria among the patients with type 2 diabetes mellitus, these two groups included a different prevalence of prior cardiovascular diseases [39]. Naka et al. suggested that the FMD was not associated with the grade of albuminuria and GFR but that it showed a negative correlation with the disease duration in the patients with type 2 diabetes mellitus [40]. It is difficult to compare the previous results with the present data, because the subjects with complicated diabetic nephropathy were excluded from their study.

Taslipinar et al. reported that the FMD was decreased in the subjects with macroalbuminuria but that it was not reduced in the subjects showing renal impairment [41]. This might be the reason for the different results from the present study, in addition to the fact that their investigation was performed in a small number (*n* = 55) of relatively younger subjects (mean age: 50 years old) with normal kidney function (GFR > 100 mL/min/1.73 m^2^). Yun et al. also reported no association between the FMD and ACR in the patients with type 2 diabetes mellitus although diabetic retinopathy was associated with a reduction of the FMD [42]. However, their study did not include subjects with macroalbuminuria, and the frequency of diabetic macroangiopathy was not described. Because the development of diabetic nephropathy and retinopathy are influenced by the duration of diabetes mellitus, the results were not considered to contradict those of our present study. Furthermore, Suetsugu et al. described the association between the FMD and all of the diabetic microangiopathies in the patients with type 2 diabetes mellitus, although the frequency of macroangiopathy was not reported [36].

The present study is associated with limitations that should be kept in mind when considering the results. First, our data do not address the causal effects of the findings, because a cross-sectional analysis was performed in the present study. We showed only that there was an association between a reduced FMD and CKD in the type 2 diabetic subjects without CHD. While it was reported that the FMD was not lower in the subjects with impaired glucose tolerance than in normal controls [15], the FMD was already disturbed in the patients with type 2 diabetes mellitus with a short duration of illness and no vascular complications [20]. Furthermore, it was described that the healthy subjects with a reduction of the FMD showed a postprandial elevation of the blood glucose level [25]. Therefore, there is no established conclusion that can be drawn about the FMD in subjects with mild hyperglycemia. Because it was reported that the postmenopausal women showing a lower FMD value frequently developed type 2 diabetes mellitus in the future [43], it is still uncertain whether the metabolic abnormalities induced by diabetes mellitus cause the reduction of the FMD or whether diabetes mellitus frequently develops in the groups of patients with endothelial dysfunction. Second, there is a problem with the diagnostic accuracy of CHD. Because CHD was defined as being present only in individuals with an obvious history of CHD or those diagnosed according to a coronary angiographic examination, subjects with asymptomatic myocardial ischemia might have been undiagnosed in the present study. This may have affected the outcome of the study, because Nguyen et al. reported that the FMD was lower in type 2 diabetic patients with asymptomatic myocardial ischemia [22].

## 6. Conclusions

The FMD was lower in the subjects with type 2 diabetes mellitus, regardless of the presence of CHD, which is one of diabetic macroangiopathies, than in those without diabetes mellitus and CHD. The stages of GFR and albuminuria were significant independent variables which were associated with the FMD in type 2 diabetic patients without CHD. Therefore, the FMD is considered to be useful for the detection of atherosclerosis in patients with type 2 diabetes mellitus, even if overt macroangiopathy is not diagnosed.

## Figures and Tables

**Table 1:** The clinical characteristics of the study subjects with and without type 2 diabetes mellitus.

	Type 2 diabetes				Nondiabetic subjects			
	Number estimated	All	CHD		Number estimated	All	CHD	
			Absent	Present			Absent	Present
		(*n* = 480)	(*n* = 319)	(*n* = 161)		(*n* = 240)	(*n* = 95)	(*n* = 145)
Age (years)	480	66 ± 12	65 ± 13	67 ± 11^∗^	240	66 ± 12	66 ± 13	66 ± 11
Male (%)	480	63	61	68	240	66	61	69
Duration of diabetes mellitus (years)	442	12 ± 12	11 ± 12	15 ± 12^∗∗^	—	—	—	—
Current smoker (%)	477	32	30	35	239	30	40	15^∗∗^
Body mass index (kg/m^2^)	480	25.6 ± 5.2	25.8 ± 5.5	25.0 ± 4.5	240	23.5 ± 4.0^##^	22.5 ± 3.5	24.1 ± 4.2^∗∗^
Obesity (%)	480	48	50	43	240	32^##^	21	39^∗∗^
Medication								
Insulin use (%)	480	33	33	32	—	—	—	—
RAS inhibitor use (%)	480	51	48	58^∗^	240	55	36	68^∗∗^
Statin use (%)	480	50	39	73^∗∗^	240	48	23	64^∗∗^
Xanthine oxidase inhibitors use (%)	480	15	13	19	240	13	12	13
Systolic blood pressure (mmHg)	479	132 ± 23	136 ± 23	125 ± 21^∗∗^	236	118 ± 20^##^	117 ± 17	118 ± 22
Diastolic blood pressure (mmHg)	479	76 ± 15	79 ± 15	70 ± 13^∗∗^	236	70 ± 15^##^	71 ± 14	70 ± 15
Hypertension (%)	480	81	77	88^∗∗^	240	89^##^	79	96^∗∗^
HbA1c (%)	449	8.8 ± 2.2	9.2 ± 2.3	8.1 ± 1.8^∗∗^	222	5.7 ± 0.5^##^	5.6 ± 0.5	5.8 ± 0.5^∗^
LDL-cholesterol (mmol/L)	473	2.83 ± 0.94	2.95 ± 0.94	2.59 ± 0.91^∗∗^	231	2.90 ± 0.94	2.62 ± 0.75	3.08 ± 1.00^∗∗^
Hyper-LDL-cholesterolemia (%)	480	54	44	74^∗∗^	240	50	25	67^∗∗^
HDL-cholesterol (mmol/L)	474	1.24 ± 0.36	1.29 ± 0.36	1.14 ± 0.35^∗∗^	228	1.22 ± 0.51^#^	1.25 ± 0.59	1.16 ± 0.36
Hypo-HDL-cholesterolemia (%)	474	30	26	39^∗∗^	228	40^##^	37	45
Serum uric acid (*μ*mol/L)	469	322 ± 99	308 ± 94	347 ± 104^∗∗^	236	358 ± 102^##^	355 ± 110	361 ± 98
Hyperuricemia (%)	479	39	36	43	239	44	35	50^∗^
Serum creatinine (*μ*mol/L)	480	102 ± 129	92 ± 111	123 ± 156^∗∗^	240	96 ± 126	91 ± 87	99 ± 146
eGFR (mL/min/1.73 m^2^)	480	69.4 ± 29.7	74.0 ± 29.8	60.3 ± 27.2^∗∗^	240	68.3 ± 23.7	67.5 ± 24.8	68.8 ± 23.2
GFR stage								
G1/G2/G3a/G3b/G4/G5 (%)	480	24/38/16/11/6/5	30/40/13/9/5/4	12/36/23/14/8/7^∗∗^	240	27/48/22/8/3/3	17/43/25/9/2/3	17/51/20/7/3/3
Albuminuria stage								
A1/A2/A3 (%)	447	45/32/23	46/34/20	42/29/29	131	60/33/8	66/26/8	51/42/7
Diabetic retinopathy (%)	378	44	40	55^∗^	—	—	—	—
Diabetic neuropathy (%)	371	60	55	73^∗∗^	—	—	—	—
ABI	455	1.11 ± 0.12	1.12 ± 0.11	1.09 ± 0.15	211	1.11 ± 0.14	1.12 ± 0.17	1.11 ± 0.13
baPWV (cm/s)	455	1731 ± 451	1727 ± 442	1740 ± 472	211	1587 ± 427^##^	1512 ± 375	1711 ± 480^∗∗^
cIMT (mm)	418	1.05 ± 0.24	1.00 ± 0.22	1.15 ± 0.26^∗∗^	165	1.01 ± 0.22	0.98 ± 0.21	1.04 ± 0.23

RAS inhibitors: renin-angiotensin system inhibitors (angiotensin-converting enzyme inhibitors or angiotensin II receptor blockers).

Xanthine oxidase inhibitors: allopurinol or febuxostat.

^∗^ *P* < 0.05 and ^∗∗^ *P* < 0.01 versus the subjects without CHD.

^#^ *P* < 0.05 and ^##^ *P* < 0.01 versus the subjects with type 2 diabetes.

**Table 2:** The relationship between the FMD and clinical parameters in the type 2 diabetic patients with and without CHD.

Parameters	CHD (−)				CHD (+)			
	Univariate		Multivariate		Univariate		Multivariate	
	Regression coefficient	*P*	Regression coefficient	*P*	Regression coefficient	*P*	Regression coefficient	*P*
Age (years)	−0.036	0.01	−0.019	0.20	−0.015	0.44		
Male (%)	−0.097	0.61			−0.018	0.94		
Duration of diabetes mellitus (years)	−0.031	0.05			−0.030	0.14		
Current smoker (%)	−0.146	0.47			−0.035	0.88		
Body mass index (kg/m^2^)	−0.026	0.43			0.049	0.32		
Obesity (%)	0.002	0.99			0.345	0.12		
Medication								
Insulin use (%)	−0.133	0.50			−0.408	0.08		
RAS inhibitor use (%)	−0.335	0.07			−0.020	0.93		
Statin use (%)	−0.231	0.23			−0.046	0.85		
Xanthine oxidase inhibitors use (%)	−0.475	0.08			0.081	0.77		
Systolic blood pressure (mmHg)	0.004	0.65			0.005	0.63		
Diastolic blood pressure (mmHg)	0.016	0.20			0.007	0.69		
Hypertension (%)	−0.133	0.55			−0.879	<0.01	−0.827	0.01
HbA1c (%)	0.143	0.09			0.016	0.90		
LDL-cholesterol (mg/dL)	0.174	0.39			0.455	0.06		
Hyper-LDL-cholesterolemia (%)	−0.127	0.50			0.005	0.98		
HDL-cholesterol (mg/dL)	0.497	0.34			1.075	0.09		
Hypo-HDL-cholesterolemia (%)	−0.008	0.97			−0.533	0.02	−0.501	0.02
Serum uric acid (mg/dL)	−0.004	0.03			−0.002	0.34		
Hyperuricemia (%)	−0.007	0.97			0.034	0.88		
Serum creatinine (mg/dL)	−0.002	0.26			−0.001	0.33		
eGFR (mL/min/1.73 m^2^)	0.022	<0.01	0.016	0.02	0.015	0.06		
Albuminuria (%)	−0.562	<0.01	−0.415	0.03	−0.449	0.07		
ABI	1.124	0.52			0.928	0.56		
baPWV (cm/s)	−0.001	0.20			−0.001	0.36		
cIMT (mm)	−0.979	0.26			−1.497	0.11		
Diabetic retinopathy (%)	−0.306	0.13			−0.027	0.42		
Diabetic neuropathy (%)	−0.261	0.19			0.043	0.89		

RAS inhibitors: renin-angiotensin system inhibitors (angiotensin-converting enzyme inhibitors or angiotensin II receptor blockers).

Xanthine oxidase inhibitors: allopurinol or febuxostat.

Albuminuria: albuminuria stage A1 or A2.
